# Effects of feeding mycotoxin-contaminated diets and the use of a yeast cell wall extracts mycotoxin adsorbent on ruminal and fecal microbiota of finishing beef steers

**DOI:** 10.3389/fmicb.2025.1675653

**Published:** 2025-10-21

**Authors:** Luis Henrique Cursino Batista, Yury Tatiana Granja-Salcedo, Igor Machado Ferreira, Mailza Gonçalves de Souza, Mateus José Inácio de Abreu, Luiz Fernando Costa e Silva, Anne Koontz, Vaughn Holder, James Eugene Pettigrew, Gustavo Rezende Siqueira, Flávio Dutra de Resende

**Affiliations:** ^1^Department of Animal Science, Faculty of Agricultural and Veterinary Sciences, São Paulo State University, Jaboticabal, Brazil; ^2^Department of Animal Science, Agência Paulista de Tecnologia dos Agronegócios (APTA), Colina, Brazil; ^3^Corporacion Colombiana de Investigacion Agropecuaria (AGROSAVIA), Centro de Investigacion el Nus, Corregimiento San Jose del Nus, San Roque, Antioquia, Colombia; ^4^Alltech, Maringa, Paraná, Brazil; ^5^Alltech Inc, Nicholasville, KY, United States; ^6^Pettigrew Research Services, Tubac, AZ, United States

**Keywords:** aflatoxins, archaea diversity, feedlot cattle, *fusarium* mycotoxins, mycotoxin binder, rumen bacteria diversity

## Abstract

**Introduction:**

This study evaluated the effects of contamination of the beef cattle diet with mycotoxins and the use of yeast cell wall extract based mycotoxin adsorbent(YCWE) on the ruminal and fecal microbial communities.

**Methods:**

Eight rumen-cannulated Nellore steers [initial body weight (BW) = 417 ± 42 kg; ± 36 month of age] were used in a 4 × 4 replicated Latin square design. A 2 × 2 factorial treatment structure was used to investigate the effects of mycotoxin contamination of the diet, the addition of YCWE and their interactions. The dietary treatments consisted of: (1) diet without mycotoxins (CTRL), and (2) control diet with added mycotoxins(MYCOT). The second factor was: (1) absence (YCWE−) or (2) presence (YCWE+)of YCWE. The addition of YCWE to the diets was 1 g/kg of dry matter (DM).

**Results:**

In the rumen, MYCOT increased microbial richness and diversity indices (*p* < 0.01), whereas YCWE decreased richness but increased diversity (*p* < 0.01). MYCOT contamination also increased the relative abundance of taxa associated with inefficient nitrogen utilization (*p* < 0.08). YCWE supplementation affected several microbial groups, reducing the abundance of methanogenic archaea and acetateproducing bacteria (*p* < 0.02). Predicted metabolic pathways indicated that MYCOT impaired several functions related to microbial growth and protein synthesis, while YCWE supplementation in contaminated diets partially restored pathways such aspurine and pyrimidine metabolism (*p* < 0.05). However, YCWE supplementation inuncontaminated diets reduced pathways linked to protein synthesis (*p* < 0.05). In feces, MYCOT and YCWE had no effects on richness (*p* > 0.10), although MYCOT increased diversity (*p* = 0.01). Treatment effects on predicted metabolic pathways of fecal microbiota were minimal, suggesting a low impact of MYCOT on fecal microorganisms (*p* > 0.10).

**Conclusion:**

Contamination of beef cattle diets with multiple mycotoxins altered ruminal and fecal microbial richness, diversity, and metabolic pathways, potentially reducing microbial growth and protein synthesis. YCWE mitigated several of these adverse effects, contributing to partial recovery of disrupted metabolic pathways. This study provides evidence that YCWE counteracts the antimicrobial effects of mycotoxins, offering a practical nutritional strategy to preserve rumen functionality.

## 1 Introduction

Mycotoxins are secondary metabolites produced by filamentous fungi under environmental stress, enhancing their pathogenicity, aggressiveness, and virulence (Venkatesh and Keller, 2019). In livestock systems, ruminants are considered more resistant to mycotoxins compared to non-ruminant animals. This resistance is partly due to the transformation of most mycotoxins into less toxic or non-toxic derivatives by microorganisms in the rumen (Fink-Gremmels, 2008; Gallo et al., 2015). However, this detoxification capacity varies significantly and depends strongly on the microbiota composition and rumen passage rate (Smith and Thakur, 2005; Fink-Gremmels, 2008). Feedlot conditions may be associated with diminished mycotoxin degradation by the rumen microbiota, due to the high concentrate level in the diet (Debevere et al., 2020). Furthermore, while the ruminal microbiota acts on mycotoxins, it does not exclude the potential antimicrobial effects of mycotoxins on microorganisms.

Studies with ruminants have hypothesized the effects of mycotoxins on the rumen microbiome may explain observations of reduced rumen fermentation (Gallo et al., 2015; Jiang et al., 2019; Hartinger et al., 2022), changes in the fermentation product profile (Jiang et al., 2019; Gallo et al., 2021; Hartinger et al., 2022; Batista et al., 2024), lower diet digestibility (Gallo et al., 2020; Batista et al., 2024), and reduced animal performance (Custodio et al., 2020; Gallo et al., 2020; Marczuk et al., 2023). In addition, under conditions of reduced ruminal pH, such as those induced by high-concentrate diets, the bioavailability of mycotoxins like aflatoxin B1 and ochratoxin A in the post-ruminal tract may increase, thereby intensifying their toxic effects (Pantaya et al., 2016). Furthermore, some mycotoxins, such as fumonisins, are predominantly excreted in the feces, due to limited ruminal degradation and/or intestinal adsorption (Smith and Thakur, 1996). In dairy cows, fumonisin exposure has been linked to greater ruminal microbial richness (Hartinger et al., 2022).

To counteract these adverse effects, different types of adsorbents have been investigated, broadly classified as inorganic (e.g., bentonite, zeolite, and activated carbon; Xu et al., 2022) and organic (e.g., yeast cell wall derivatives rich in *β*-glucans and mannan oligosaccharides; Zhu et al., 2016). Inorganic adsorbents are generally more effective against aflatoxins but show limited binding capacity for other mycotoxins (Vila-Donat et al., 2018), whereas organic adsorbents exhibit a broader spectrum, binding to deoxynivalenol, zearalenone, and ochratoxin A, with positive outcomes reported in both *in vitro* (Yiannikouris et al., 2006; Cavret et al., 2010; Pfohl-Leszkowicz et al., 2015) and *in vivo* studies (Díaz-Llano and Smith, 2007; Weaver et al., 2014; Pfohl-Leszkowicz et al., 2015). However, despite this evidence, limited information is available on how multiple mycotoxins, under practical farm-level concentrations, simultaneously affect both the ruminal and fecal microbiota and their predicted metabolic functionality. In this context, the novelty of this study lies in its integrated assessment of the rumen-fecal microbial community and predicted microbial functions, providing new insights into the mechanisms by which mycotoxins and the use of YCWE impact microbial ecology and beef cattle productive efficiency.

When feed ingredients are contaminated with mycotoxins, the inclusion of a mycotoxin adsorbent composed of yeast cell wall extract (*Saccharomyces cerevisiae*) in cattle diets has been used to mitigate the negative effects of mycotoxins on animal performance (Custodio et al., 2020; Xu et al., 2020, 2022). Mycotoxin sequestering agents are compounds that bind mycotoxins in contaminated feeds without dissociating, enabling their elimination via feces as they pass through the gastrointestinal tract. The major functional components of yeast cell wall responsible for mycotoxin binding are *β*-D-glucan and mannan oligosaccharides, which bind to mycotoxins through hydrogen bonding and van-der-Waal forces (Yiannikouris et al., 2006; Jouany, 2007).

Additionally, Zhou et al. (2023) demonstrated that yeast cell-wall polysaccharides-based additives can modulate gut microbial composition by promoting the proliferation of beneficial microbes and suppressing the colonization of pathogens. We hypothesized that mycotoxin contamination (MYCOT) at levels which may be realistically encountered in feedlot situations (Custódio et al., 2019; Biscoto et al., 2022) would disturb the rumen and fecal microbiome, and that the use of a mycotoxin adsorbent (YCWE) could help mitigate some of the negative impacts of mycotoxin contamination. Therefore, the objectives of this study were to investigate the effects of MYCOT, YCWE and their interaction (MYCOT × YCWE) on the ruminal and fecal microbial communities of finishing beef steers.

## 2 Materials and methods

### 2.1 Animals, feeding and treatments

All experimental procedures in this study were carried out in accordance with the ethical principle established by the Brazilian Council for the Control of Animal Experimentation and approved by the Ethics Committee for the Use of Animals of the Department of Development Decentralization, São Paulo, Brazil (protocol #0005/2020).

Animal management, experimental design, and treatments were previously described in detail (Batista et al., 2024). Briefly, 8 rumen-cannulated Nellore steers [initial body weight (BW) = 417 ± 42 kg; ± 36 month of age] were used in a 4 × 4 duplicate Latin square design. The experiment had four 29-d periods including 21 d of adaptation to the experimental treatments, 8 d of sampling, and one-week washout between each period to minimize carryover effects in the next period (Hartinger et al., 2022).

The basal diet without added mycotoxins and without adsorbent was the same for all treatments and was provided during the washout period. The steers were fed twice daily (08:00 and 15:00 h) *ad libitum* a total mixed ration (TMR) consisting of 12% sugarcane bagasse as a roughage source, 61.8% dry ground corn, 15% citrus pulp, 8% soybean meal, and 3.2% of a feedlot premix (Every kilogram of premix contained 131 g Ca, 15.3 g P, 41 g S, 17.1 g Mg, 53 g Na, 451 mg Cu, 1489 mg Zn, 462 mg Mn, 43 mg I, 59 mg Co, 6 mg Se, 451 mg Fe, 106 mg F, 96,770 IU vitamin A, 800 mg monensin; Non-protein nitrogen (NPN) - equivalent to crude protein (CP) 108.4%, CP 111.2%).

The treatments were designed to evaluate the effects of contamination of multiple mycotoxins at concentrations found in practical conditions in beef cattle diets (Custódio et al., 2019; Biscoto et al., 2022), and the use of a mycotoxin adsorbent based on yeast cell wall extract (Custodio et al., 2020). Therefore, the treatments were evaluated in a 2 × 2 factorial arrangement, where the first factor was to evaluate mycotoxin contamination: (1) diets without added mycotoxins (CTRL) and (2) diets with added mycotoxin contamination (MYCOT), and the second factor to evaluate the use of a yeast cell wall extract mycotoxin adsorbent (YCWE): (1) diets without added YCWE (YCWE−) and (2) diets with added YCWE (YCWE+; Mycosorb A^+^, Alltech, Nicholasville, KY, USA). Therefore, four treatments were evaluated: (1) CTRL YCWE−, (2) CTRL YCWE+, (3) MYCOT YCWE−, (4) MYCOT YCWE+. Mycosorb A^+^ is an organic mycotoxin adsorbent composed of parietal components of *Saccharomyces cerevisiae* and algae. The inclusion of YCWE in diet was 1.0 g/kg of DM.

The mycotoxin contamination procedure and analysis of mycotoxins in feed have been previously described in detail (Batista et al., 2024). Briefly, the dose of mycotoxins used in this study were based on a survey previously conducted by Custódio et al. (2019). The mycotoxins used in this study (Aflatoxins B1 + B2, Fumonisins B1 + B2 + B3, Trichothecenes B [mainly DON], Zearalenone, and Roquefortine C) were produced individually at the University of São Paulo through natural fermentation of corn or wheat by specific fungal species. Mycotoxin concentrations were standardized and added daily to the feed of cattle assigned to the MYCOT treatment. In general, the CTRL diets had natural contamination of 877.1 μg/kg dry matter basis of fumonisins, 53.8 fusaric acid and 75.7 emerging mycotoxins (beauvericin + moniliformin). MYCOT diets had 12.2 μg/kg aflatoxins, 4544.4 fumonisins, 1423.4 trichothecenes B (DON (Deoxynivalenol), 15-acetyl DON, 3-acetyl DON), fusarenol 18.4 Roquefortine C, 87.6 emerging mycotoxins.

### 2.2 Ruminal and fecal sample collection

Ruminal and fecal samples were collected on d 22 of each experimental period, early in the morning, before the morning feeding (Saro et al., 2012). For rumen microbiota assessment, samples weighing approximately 50 g per animal (a mix of liquid and solid) were collected through the ruminal cannula, immediately placed into cryotubes and frozen in liquid nitrogen, and subsequently, stored at −80 °C until further DNA extraction.

Feces (approximately 400 g wet) were collected via the rectum of each animal before feeding. The collected fecal samples were mixed well and subsampled, with 2 g of feces frozen in liquid nitrogen and stored at −80 °C until subsequent DNA extraction.

### 2.3 DNA extraction, high-throughput sequencing, and data analysis

Total DNA was extracted using Quick-DNA Fecal/Soil Microbe Kits (Zymo Research, Murphy Ave Irvine, CA 92614, EUA) according to the manufacturer’s instructions. The quality and quantity of extracted DNA were measured using a NanoDrop 1000 spectrophotometer (NanoDrop Technologies Inc., Wilmington, DE). Duplicate libraries were prepared by polymerase chain reaction (PCR) amplification of the V3 and V4 regions of the 16S ribosomal RNA gene (16S rRNA) for bacteria using the universal primers 515F and 806R as described by Caporaso et al. (2010). Each PCR reaction mixture contained 20 ng of metagenomic DNA, 10μM of each forward and reverse primers, 1.25 mM of magnesium chloride, 200μM of dNTP mix (Invitrogen, Carlsbad, CA, USA), 1.0 U Platinum Taq DNA polymerase high fidelity (Invitrogen, Carlsbad, CA, USA), high fidelity PCR buffer [1X], and milli-Q water. Reactions were held at 95 °C for 3 min, with amplification proceeding for 30 cycles at 95 °C for 30 s, 53.8 °C for 30 s, and 72 °C for 45 s; a final extension of 10 min at 72 °C was added to ensure complete amplification. The expected fragment length of PCR products was verified by agarose gel (1%) electrophoresis, and the amplicon size was estimated by comparison with a 1 kb plus DNA ladder (1 kb plus DNA ladder, Invitrogen, Carlsbad, CA, USA). The PCR fragments were purified using the ZymocleanTM Gel DNA Recovery kit following the manufacturer’s instructions.

All sequence data were processed, removing adapters using Scythe 0.991^1^ and Cutadapt 1.7.1 (Martin, 2011). Sequence trimming was carried out by selecting sequences ~470 bp in length with an average quality score higher than 40 based on Phred quality, and duplicate reads were removed using the Prinseq program (Schmieder and Edwards, 2011). The QIIME software package version 1.9.1 was used to filter reads and determine Operational Taxonomic Units (OTUs) as described in Caporaso et al. (2010). Significant readings were classified based on the multinomial naive Bayes algorithm to group the OTUs of readings with a cut of 98% and, to assign the taxonomy, the SILVA Ribosomal Database Project (RDP-II) was used. Bacterial sequences were de-noised, and suspected chimeras were removed using the OTU pipe function within QIIME. Sequence data were summarized at the phylum, class, and family levels; Also, Alpha_diversity.py in QIIME was used to calculate ACE, Chao1, Shannon, and Simpson indices. Microbial function prediction for each ruminal and fecal sample based on 16S rRNA gene sequencing data was determined using Phylogenetic Investigation of Communities by Reconstruction of Unobserved States (PICRUST; Wilkinson et al., 2018).

The apparent relative abundance of microbial communities, diversity indices and relative predicted microbial function abundances were analyzed using R Software version 4.3.3 (R Core Team, 2023). Because the data did not meet assumptions of normality and homogeneity of variances (Shapiro–Wilk and Levene tests), the non-parametic statistical test, Friedman test, was applied to compare the factors: Mycotoxin contamination (CTRL vs. MYCOT) and use of YCWE (YCWE− vs. YCWE+) and Kruskal-Wallis test for comparison between treatments resulting from the M × Y interaction (CTRL YCWE−, CTRL YCWE+, MYCOT YCWE− and MYCOT YCWE+). Significance was set at *p* ≤ 0.05, and trends were determined if *p* > 0.05 and ≤ 0.10.

## 3 Results

### 3.1 Taxonomic composition of the ruminal microbiota

**Figure 1:**
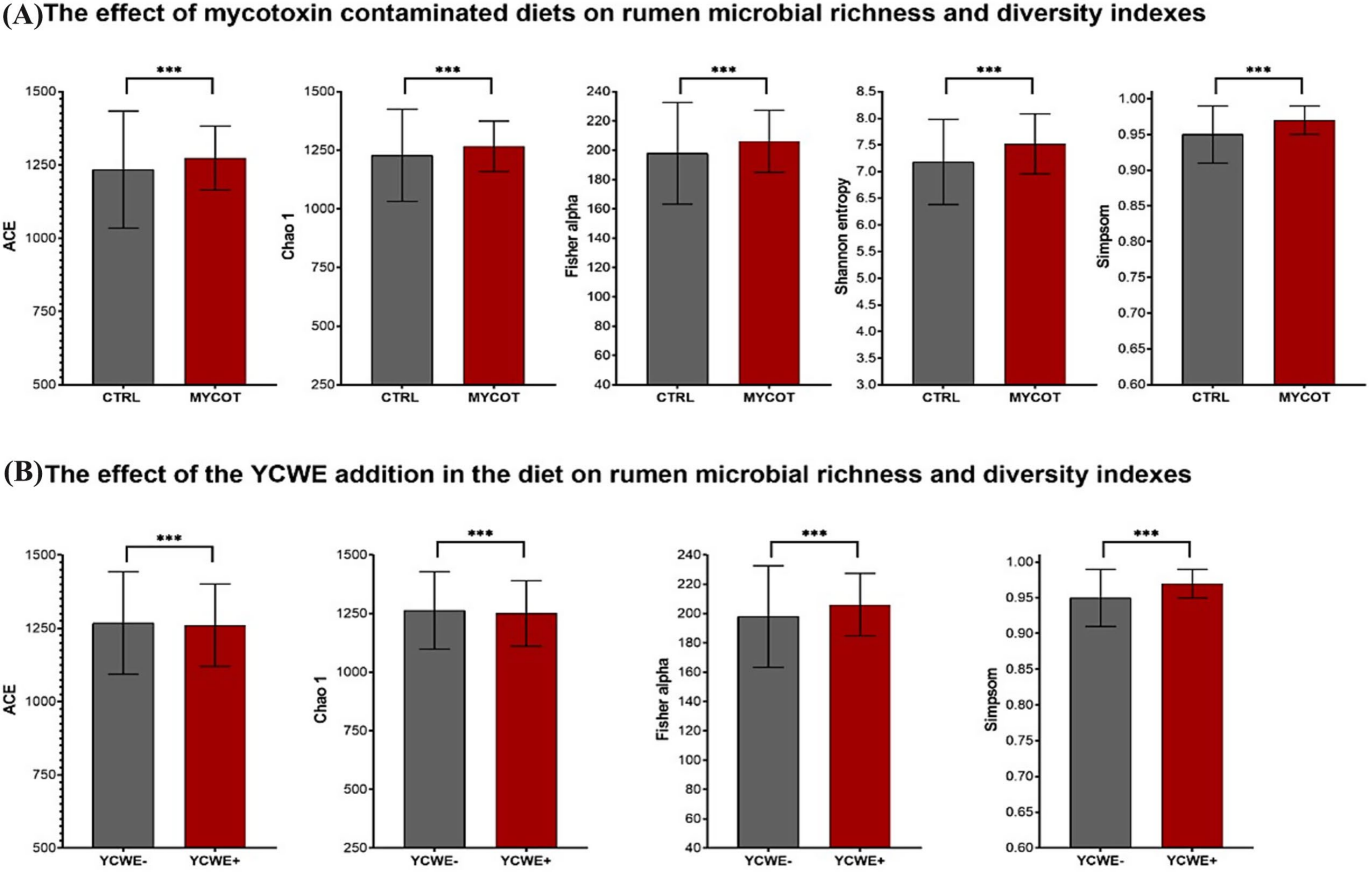
Median and interquartile range of microbial richness and diversity indexes for the rumen in beef steer fed control (CTRL) or mycotoxin contaminated (MYCOT) diets **(A)**, with the addition or not of an absorbent YCWE in the diet **(B)**. *** significative differences (*p* < 0.05) based on Friedman’s test.

In the rumen microbiota, both factors (MYCOT and YCWE) significantly affected (*p* = 0.01) the richness indices (ACE and Chao1) and the diversity indices (Fisher’s alpha and Simpson; Table 1; Figure 1). However, YCWE had no effect on Shannon entropy (*p* = 0.90). Diet contamination with MYCOT increased richness and diversity (p = 0.01), whereas YCWE supplementation decreased richness but increased diversity (*p* = 0.01; Figure 1). No MYCOT × YCWE interaction was detected for any richness or diversity index (*p* ≥ 0.26).

**Table 1:** Median and interquartile range of microbial richness and diversity indexes for the rumen and fecal environments in beef steers-fed mycotoxin contaminated diets (MYCOT) and the use of mycotoxin adsorbent (YCWE).

Item	CTRL		MYCOT		*p*-value		
	YCWE−	YCWE+	YCWE−	YCWE+	MYCOT	YCWE	M × Y
Rumen							
Bacteria %	96.7 ± 2.97	98.3 ± 2.60	97.7 ± 1.44	98.1 ± 1.75	0.95	0.32	0.55
Archaea %	3.32 ± 2.94	1.69 ± 2.62	2.34 ± 1.43	1.89 ± 1.75	0.95	0.32	0.55
Richness							
ACE	1234 ± 236	1244 ± 254	1274 ± 170	1261 ± 148	0.01	0.01	0.92
Chao 1	1228 ± 229	1243 ± 253	1271 ± 162	1250 ± 748	0.01	0.01	0.90
Diversity							
Fisher alpha	196 ± 44	201 ± 51	209 ± 32	202 ± 16	0.01	0.01	0.88
Shannon entropy	7.17 ± 0.47	7.31 ± 0.92	7.52 ± 0.63	7.50 ± 0.56	0.01	0.90	0.26
Simpson	0.95 ± 0.02	0.96 ± 0.03	0.97 ± 0.01	0.97 ± 0.01	0.01	0.01	0.33
Feces							
Bacteria %	87.0 ± 3.03	86.3 ± 8.00	84.4 ± 7.05	86.7 ± 5.97	0.95	0.83	0.99
Archaea %	13.04 ± 3.04	13.71 ± 8.00	15.6 ± 7.06	13.3 ± 5.98	0.95	0.83	0.99
Richness							
ACE	1390 ± 399	1477 ± 293	1404 ± 281	1453 ± 261	0.91	0.99	0.95
Chao 1	1382 ± 409	1468 ± 300	1401 ± 289	1442 ± 249	0.99	0.99	0.94
Diversity							
Fisher alpha	230 ± 76	254 ± 61	234 ± 49	247 ± 56	0.99	0.90	0.92
Shannon entropy	8.29 ± 0.15	8.55 ± 0.34	8.13 ± 0.42	8.11 ± 0.66	0.01	0.01	0.21
Simpson	0.95 ± 0.02	0.99 ± 0.01	0.98 ± 0.01	0.98 ± 0.01	0.01	0.99	0.48

MYCOT = effect of mycotoxin contamination diets as control or Mycotoxins evaluated by a Friedman test. YCWE = effect of the addition or not of an adsorbent in the diet evaluated by a Friedman test. M × Y = Effect of mycotoxin contamination and addition of an adsorbent interaction evaluated by a Kruskal-Wallis test.

**Figure 2:**
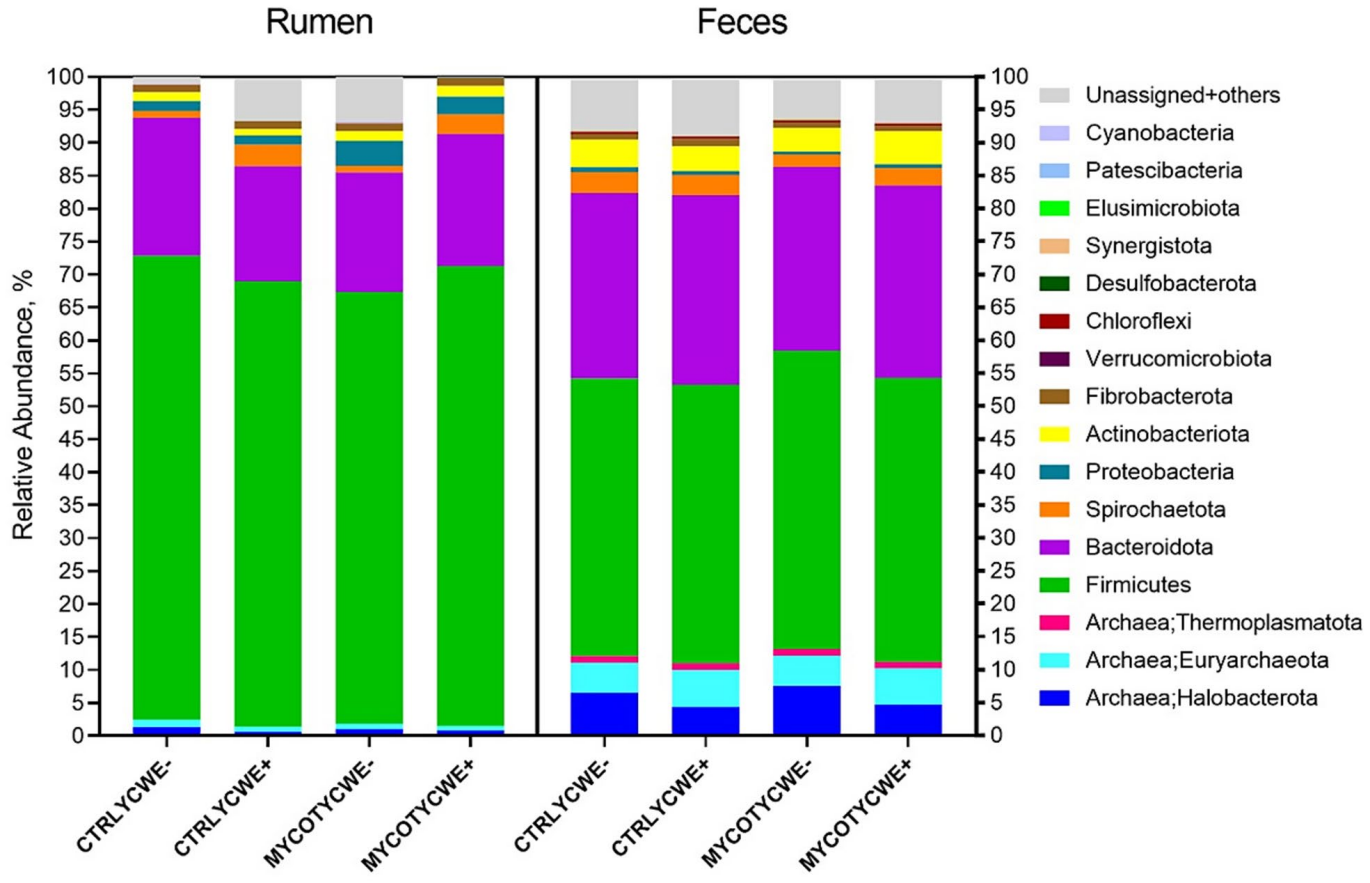
Relative abundance of bacteria and archaea phyla identified for the rumen and fecal environments in beef steers-fed control (CTRL) or mycotoxin contaminated diets (MYCOT) associated or not to the use of an adsorbent (YCWE). Others = summatory of Myxococcota, Bdellovibrionota, Planctomycetota, Deferrisomatota, Nitrospirota, Deinococcota, WPS-2, Sva0485, Gemmatimonadota, TA06, Fusobacteriota, Campilobacterota, Methylomirabilota in fecal samples and summatory of Nitrospirota, Gemmatimonadota, WPS-2, Dependentiae, Bdellovibrionota, Myxococcota, Armatimonadota, Planctomycetota, Campilobacterota, Latescibacterota, Methylomirabilota, Entotheonellaeota, Deferrisomatota, RCP2-54, NB1-j, Fusobacteriota, Zixibacteria, MBNT15, Deinococcota, WS2, Abditibacteriota, and NKB15 in rumen samples.

The relative abundance of total bacteria and total archaea were not different between treatments (*p* ≥ 0.32). There was also no effect (*p* ≥ 0.21) on the predominant phyla (Firmicutes, Bacteroidota, Spirochaetota, Proteobacteria, Actinobacteriota; Figure 2). However, the phylum Desulfobacterota tended (*p* = 0.06) to have higher abundance in the rumen of steers fed MYCOT with YCWE addition (0.032 ± 0.030) when compared to observed in steers fed CTRL diet without YCWE addition (0.010 ± 0.020). In the CTRL YCWE+ and MYCOT YCWE− treatments, more unassigned + others OTUs were observed (Figure 2).

At the class level, only the Clostridia tended to be greater in the rumen of steers fed MYCOT diets (52.12 ± 11.7%) when compared to those fed Control diets (51.60 ± 7.46%; *p* = 0.08). The most pronounced MYCOT-induced changes at the family level were the trend towards higher abundance of Lachnospiraceae (*p* = 0.10) and lower abundance of Christensenellaceae (*p* = 0.10; Table 2). With the use of YCWE, we observed a greater abundance of Butyricicoccaceae (*p* = 0.01) and a tendency towards a greater abundance of Prevotellaceae (*p* = 0.08), as well as lower abundance of Succinivibrionaceae (*p* = 0.05) and the archaea family Methanomicrobiaceae (*p* = 0.02).

**Table 2:** Median and interquartile range of the rumen relative abundance (%) of methanogens and bacteria at family level in beef steers-fed mycotoxin contaminated diets and the use of an adsorbent (YCWE).

Domain	Phylum	Family	CTRL		MYCOT		*p*-value		
			YCWE−	YCWE+	YCWE−	YCWE+	MYCOT	YCWE	M × Y
Bacteria	Actinobacteriota	Pseudonocardiaceae	0.010 ± 0.0^b^	0.020 ± 0.02^b^	0.050 ± 0.01^a^	0.020 ± 0.01^ab^	0.32	0.53	0.06
	Firmicutes	Lachnospiraceae	12.8 ± 9.88	11.2 ± 4.89	14.9 ± 9.37	16.1 ± 8.09	0.10	0.40	0.26
		Acidaminococcaceae	0.585 ± 0.37^ab^	0.380 ± 0.24^b^	0.625 ± 0.16^a^	0.705 ± 0.30^a^	0.17	0.50	0.10
		Christensenellaceae	1.53 ± 0.75	1.56 ± 0.86	1.07 ± 0.42	0.940 ± 0.53	0.10	0.87	0.20
		Enterococcaceae	0.000 ± 0.01^b^	0.105 ± 0.28^a^	0.190 ± 0.33^a^	0.015 ± 0.13^b^	0.31	0.97	0.10
		Butyricicoccaceae	0.040 ± 0.05	0.075 ± 0.09	0.050 ± 0.01	0.075 ± 0.14	0.65	0.01	0.47
		Peptostreptococcales-Tissierellales	0.010 ± 0.02^a^	0.001 ± 0.02^b^	NI^c^	NI^c^	0.31	0.89	0.04
	Proteobacteria	Succinivibrionaceae	0.820 ± 0.83	0.490 ± 0.39	1.32 ± 1.79	0.730 ± 0.62	0.99	0.05	0.17
		Pectobacteriaceae	0.000 ± 0.02^b^	0.075 ± 0.18^ab^	0.325 ± 0.55^a^	0.095 ± 0.16^b^	0.31	0.55	0.01
	Bacteroidota	Prevotellaceae	0.160 ± 0.12	0.180 ± 0.23	0.185 ± 0.36	0.270 ± 0.32	0.31	0.08	0.69
		Bacteroidales UCG-001	0.250 ± 0.02^a^	0.010 ± 0.02^ab^	0.000 ± 0.01^b^	0.050 ± 0.02^ab^	0.60	0.37	0.07
Archaea	Halobacterota	Methanomicrobiaceae	1.34 ± 1.60	0.580 ± 0.37	0.995 ± 1.79	0.815 ± 0.88	0.31	0.02	0.20

MYCOT = effect of mycotoxin contamination diets as control or Mycotoxins evaluated by a Friedman test. YCWE = effect of the addition or not of an adsorbent in the diet evaluated by a Friedman test. M × Y = Effect of mycotoxin contamination and addition of an adsorbent interaction evaluated by a Kruskal-Wallis test. NI = not identified OTU. ^a-c^ Means 62 followed by different letters differ at *p* ≤ 0.10.

In six bacterial families, an effect or trend (*p* ≤ 0.10) of MYCOT × YCWE interaction was observed (Table 2). The abundance of Pseudonocardiaceae tended to be lower (*p* = 0.06) in CTRL diets with or without YCWE when compared to the MYCOT YCWE−. The abundance of Pectobacteriaceae was greater (*p* = 0.01) in MYCOT YCWE− compared to CTRL YCWE− and MYCOT YCWE+ treatments. Peptostreptococcales-Tissierellales were not detected in diets with mycotoxins and had greater (*p* = 0.04) abundance in the CONT YCWE− diet. Bacteroidales UCG-001 tended to be more abundant (*p* = 0.07) in CTRL YCWE− compared to MYCOT YCWE−.

**Figure 3:**
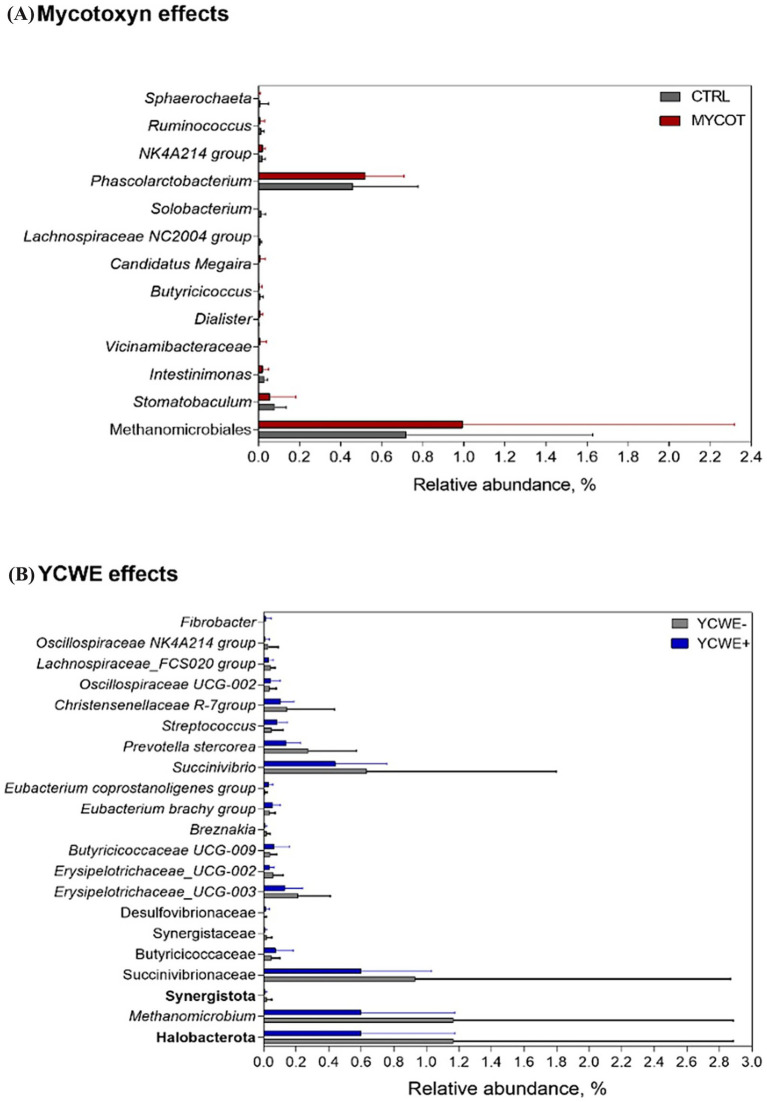
Relative abundance of differential ruminal bacteria and archaea taxa in beef steer fed control (CTRL) or mycotoxin contaminated (MYCOT) diets **(A)**, with the addition or not of an absorbent YCWE in the diet **(B)**. Only significative (*p* < 0.05) or tendencies (*p* < 0.10) values based on Friedman’s test are shown.

At the genus level (Table 3), the mycotoxin contamination factor had a tendency (*p* = 0.06) towards lower abundance of *Phascolarctobacterium* and a tendency toward greater *Dialister* (Figure 3). The use of YCWE resulted in effects or trends for lower abundance of eight genera: *Dorea* (*p* = 0.07), *Sellimonas* (*p* = 0.10), *Christensenellaceae* (*p* = 0.07), *Breznakia* (*p* = 0.04), *Muribaculaceae* (*p* = 0.09), *Prevotella* (*p* = 0.05), *Succinivibrio* (*p* = 0.07) and *Methanomicrobium* (*p* = 0.02). Rumen effects grouped by MYCOT or YCWE on other taxa are presented in Figure 3. MYCOT × YCWE interaction effects were observed on the relative abundance of the genera *Agathobacter* (*p* = 0.02), *Tepidibacter* (*p* = 0.07), *Enterococcus* (*p* = 0.08), *Erysipelotrichaceae* UCG 003 (*p* = 0.03), *Subdoligranulum* (*p* = 0.10), Breznakia (*p* = 0.08) *Dickeya* (*p* = 0.01).

**Table 3:** Median and interquartile range of the relative abundance (%) of rumen methanogens and bacteria at genera level in beef steers-fed mycotoxin contaminated diets and the use of an adsorbent (YCWE).

Domain	Phylum	Genera	CTRL		MYCOT		*p*-value		
			YCWE−	YCWE+	YCWE−	YCWE+	MYCOT	YCWE	M × Y
Bacteria	Firmicutes	*Agathobacter*	0.760 ± 0.91^c^	0.975 ± 0.78^bc^	1.96 ± 1.40^a^	1.88 ± 1.42^ab^	0.31	0.67	0.02
		*Bacillus*	0.000 ± 0.20	0.735 ± 0.96	0.595 ± 1.16	0.015 ± 0.41	0.10	0.86	0.22
		*Tepidibacter*	0.130 ± 0.07^b^	0.160 ± 0.03^ab^	0.190 ± 0.10^a^	0.255 ± 0.11^a^	0.31	0.36	0.07
		*Frisingicoccus*	0.460 ± 0.13	0.380 ± 0.20	0.365 ± 0.13	0.365 ± 0.20	0.10	0.17	0.64
		*Enterococcus*	0.00 ± 0.00^b^	0.040 ± 0.27^ab^	0.190 ± 0.26^a^	0.015 ± 0.12^ab^	0.17	0.80	0.08
		*Erysipelotrichaceae UCG 003*	0.175 ± 0.23^ab^	0.105 ± 0.04^b^	0.285 ± 0.18^a^	0.205 ± 0.13^ab^	0.65	0.07	0.03
		*Dorea*	0.225 ± 0.26	0.100 ± 0.06	0.160 ± 0.07	0.120 ± 0.04	0.31	0.07	0.43
		*Subdoligranulum*	0.120 ± 0.07^b^	0.125 ± 0.12^b^	0.185 ± 0.24^ab^	0.335 ± 0.24^a^	0.31	0.85	0.10
		*Sellimonas*	0.150 ± 0.11	0.110 ± 0.06	0.115 ± 0.12	0.100 ± 0.03	0.31	0.10	0.44
		*Phascolarctobacterium*	0.490 ± 0.42	0.305 ± 0.23	0.520 ± 0.15	0.520 ± 0.21	0.06	0.40	0.20
		*Christensenellaceae*	0.285 ± 0.29	0.105 ± 0.19	0.070 ± 0.20	0.110 ± 0.07	0.65	0.07	0.37
		*Breznakia*	0.020 ± 0.04^a^	0.010 ± 0.01^b^	0.020 ± 0.01^ab^	0.010 ± 0.01^b^	0.31	0.04	0.08
	Bacteroidota	*Muribaculaceae*	5.95 ± 2.58	3.70 ± 2.00	3.34 ± 2.01	3.87 ± 2.95	0.31	0.09	0.15
		*Prevotella*	0.215 ± 0.51	0.140 ± 0.07	0.275 ± 0.13	0.155 ± 0.09	0.65	0.05	0.37
	Proteobacteria	*Succinivibrio*	0.645 ± 0.81	0.355 ± 0.37	1.19 ± 1.67	0.595 ± 0.52	0.96	0.07	0.14
		*Dickeya*	0.000 ± 0.02^b^	0.075 ± 0.18^b^	0.325 ± 0.55^a^	0.095 ± 0.16^b^	0.31	0.55	0.01
Archaea	Halobacterota	*Methanomicrobium*	1.34 ± 1.60	0.580 ± 0.37	0.995 ± 1.79	0.815 ± 0.88	0.17	0.02	0.20

MYCOT = effect of mycotoxin contamination diets as control or Mycotoxins evaluated by a Friedman test. YCWE = effect of the addition or not of an adsorbent in the diet evaluated by a Friedman test. M × Y = Effect of mycotoxin contamination and addition of an adsorbent interaction evaluated by a Kruskal-Wallis. ^a-c^ Means 62 followed by different letters differ at *p* ≤ 0.10.

### 3.2 Taxonomic composition of the fecal microbiota

There were no differences among treatments on the richness indexes of the microbiota population in feces (*p* ≥ 0.91; Table 1). Contamination with MYCOT resulted in lower Shannon entropy and Simpson diversity indices (*p* = 0.01). The use of YCWE resulted in greater microbial diversity by the Shannon entropy index (*p* = 0.01). The Gammaproteobacteria class abundance tended to be lower in feces of steers fed a diet contaminated with MYCOT (TRL = 0.611 ± 0.53 vs. MYCOT = 0.485 ± 0.27; *p* = 0.08).

At the family level (Table 4), MYCOT had lower abundance of p-251-o5 (*p* = 0.02) and tended to lower abundance of Streptococcaceae and Succinivibrionaceae (*p* = 0.07). The use of YCWE tended (*p* = 0.07) to lower abundance of Muribaculaceae. The Morganellaceae family had lower abundance in the MYCOT YCWE− treatment and higher abundance in the CTRL YCWE+ treatment (*p* = 0.05).

**Table 4:** Median and interquartile range of the fecal relative abundance (%) of bacteria at family level in beef steers-fed mycotoxin contaminated diets and the use of an adsorbent (YCWE).

Domain	Phylum	Family	CTRL		MYCOT		*p*-value		
			YCWE−	YCWE+	YCWE−	YCWE+	MYCOT	YCWE	M × Y
Bacteria	Bacteroidota	p-251-o5	0.124 ± 0.31	0.227 ± 0.28	0.116 ± 0.02	0.106 ± 0.08	0.02	0.64	0.52
		Muribaculaceae	0.227 ± 0.49	0.254 ± 0.35	0.444 ± 0.29	0.226 ± 0.24	0.46	0.07	0.47
	Firmicutes	Streptococcaceae	0.117 ± 0.04	0.059 ± 0.06	0.044 ± 0.17	0.065 ± 0.06	0.07	0.87	0.13
	Proteobacteria	Succinivibrionaceae	0.404 ± 0.17	0.369 ± 0.14	0.256 ± 0.12	0.271 ± 0.11	0.07	0.53	0.25
		Morganellaceae	1.20 ± 0.55^ab^	1.23 ± 0.49^a^	0.873 ± 0.26^b^	1.22 ± 0.72^ab^	0.11	0.07	0.05

MYCOT = effect of mycotoxin contamination diets as control or Mycotoxins evaluated by a Friedman test. YCWE = effect of the addition or not of an adsorbent in the diet evaluated by a Friedman test. M × Y = Effect of mycotoxin contamination and addition of an adsorbent interaction evaluated by a Kruskal-Wallis test. ^a-c^ Means 62 followed by different letters differ at *p* ≤ 0.10.

At the genus level (Table 5), MYCOT had lower abundance of *Megasphaera*, *Bacteroidales_RF16_group* and the archaea *Methanosphaera* (*p* ≤ 0.03), and tended to lower abundance of *Pseudobutyrivibrio, Streptococcus, Subdoligranulum, Roseburia, Prevotellaceae_Ga6A1* (*p* ≤ 0.09). YCWE tended to have greater abundance of the genera *Christensenellaceae_R-7* (*p* = 0.06), *Clostridia_UCG-014* (*p* = 0.07) and *F082* (*p* = 0.08).

**Table 5:** Median and interquartile range of the fecal relative abundance (%) of bacteria at genera level in beef steers-fed mycotoxin contaminated diets and the use of an adsorbent (YCWE).

Domain	Phylum	Genera	CTRL		MYCOT		*p*-value		
			YCWE−	YCWE+	YCWE -	YCWE+	MYCOT	YCWE	M × Y
Bacteria	Firmicutes	*Christensenellaceae_R-7_group*	1.06 ± 1.63	1.14 ± 1.55	1.13 ± 1.12	1.99 ± 0.99	0.32	0.06	0.57
		*Clostridia_UCG-014*	0.156 ± 0.16	0.299 ± 0.25	0.211 ± 0.07	0.260 ± 0.13	0.53	0.07	0.40
		*Pseudobutyrivibrio*	0.141 ± 0.18	0.283 ± 0.37	0.106 ± 0.18	0.142 ± 0.09	0.09	0.35	0.85
		*Streptococcus*	0.118 ± 0.04	0.059 ± 0.06	0.045 ± 0.04	0.066 ± 0.06	0.07	0.83	0.13
		*Subdoligranulum*	0.044 ± 0.10	0.021 ± 0.11	0.007 ± 0.18	0.004 ± 0.02	0.09	0.63	0.50
		*Megasphaera*	0.010 ± 0.01	0.070 ± 0.01	0.012 ± 0.01	NI	0.02	0.64	0.12
		*Thermoactinomyces*	0.053 ± 0.03	0.066 ± 0.03	0.062 ± 0.05	0.093 ± 0.09	0.03	0.30	0.83
		*Roseburia*	0.028 ± 0.09	0.040 ± 0.08	0.019 ± 0.04	0.014 ± 0.04	0.07	0.34	0.67
	Bacteroidota	*Bacteroidales_RF16_group*	0.479 ± 0.39	0.535 ± 0.14	0.417 ± 0.23	0.324 ± 0.08	0.03	0.56	0.18
		*Prevotellaceae_Ga6A1_group*	0.143 ± 0.13	0.145 ± 0.22	0.109 ± 0.04	0.104 ± 0.04	0.07	0.83	0.67
		*Rikenellaceae_RC9_gut_group*	0.120 ± 0.05^ab^	0.137 ± 0.13^ab^	0.186 ± 0.11^a^	0.100 ± 0.04^b^	0.83	0.53	0.02
		*F082*	0.017 ± 0.06	0.024 ± 0.41	0.018 ± 0.11	0.088 ± 0.16	0.87	0.08	0.52
Archaea	Euryarchaeota	*Methanosphaera*	0.064 ± 0.06	0.025 ± 0.14	0.017 ± 0.02	0.022 ± 0.02	0.01	0.87	0.10

MYCOT = effect of mycotoxin contamination diets as control or Mycotoxins evaluated by a Friedman test. YCWE = effect of the addition or not of an adsorbent in the diet evaluated by Friedman test. M × Y = Effect of mycotoxin contamination and addition of an adsorbent interaction evaluated by a Kruskal-Wallis test. NI = not identified OTU. ^a-c^ Means 62 followed by different letters differ at *p* ≤ 0.10.

### 3.3 Effect of MYCOT and YCWE treatments on functional metagenome prediction

Functional metagenome prediction was used to assess potential alterations in bacterial metabolism caused by the dietary MYCOT and YCWE (Table 6). In the rumen, there were effects or trends of MYCOT × YCWE interaction (*p* ≤ 0.10) on some metabolic pathways that may be related to microbial protein synthesis in the rumen. Relative abundance of Ribosome metabolism was higher (*p* = 0.05) in the CTRL YCWE− treatment compared to the other treatments. The same pattern of results was observed for the metabolic pathways of Aminoacyl-Trna biosynthesis (*p* = 0.03) and Translation proteins (*p* = 0.07). Translation factors, Purine and Pyrimidine metabolism were higher (*p* = 0.05) in the CTRL YCWE− treatment and lower in the MYCOT YCWE− treatment.

**Table 6:** Median and interquartile range of pathways relative abundance (%) of the third-level KEGG computed for the rumen and feces in beef steers-fed mycotoxin contaminated diets and the use of an adsorbent (YCWE).

Rumen	CTRL		MYCOT		*p*-value		
	YCWE−	YCWE+	YCWE−	YCWE+	MYCOT	YCWE	M × Y
Transporters	6.21 ± 0.16	6.18 ± 0.14	6.19 ± 0.213	6.15 ± 0.35	0.10	0.73	0.94
DNA repair and recombination proteins	3.19 ± 0.11	3.15 ± 0.09	3.14 ± 0.09	3.15 ± 0.11	0.08	0.25	0.32
Ribosome	2.83 ± 0.10^a^	2.79 ± 0.12^b^	2.71 ± 0.113^b^	2.74 ± 0.10^b^	0.86	0.28	0.05
Purine metabolism	2.60 ± 0.06^a^	2.58 ± 0.10^ab^	2.54 ± 0.037^b^	2.56 ± 0.03^ab^	0.09	0.57	0.05
Pyrimidine metabolism	2.18 ± 0.05^a^	2.16 ± 0.11^ab^	2.10 ± 0.08^b^	2.13 ± 0.07^ab^	0.31	0.50	0.05
Two-component system	1.33 ± 0.11^b^	1.38 ± 0.13^ab^	1.46 ± 0.05^a^	1.40 ± 0.11^ab^	0.65	0.66	0.04
Aminoacyl-Trna biosynthesis	1.33 ± 0.03^a^	1.30 ± 0.04^b^	1.27 ± 0.035^b^	1.29 ± 0.03^b^	0.59	0.31	0.03
Secretion system	1.23 ± 0.03^b^	1.26 ± 0.05^ab^	1.29 ± 0.015^a^	1.26 ± 0.06^a^	0.31	0.52	0.02
Translation proteins	1.00 ± 0.02^a^	0.98 ± 0.03^b^	0.97 ± 0.023^b^	0.98 ± 0.01^b^	0.65	0.29	0.07
Bacterial motility proteins	1.00 ± 0.17	1.01 ± 0.19	1.15 ± 0.12	1.07 ± 0.12	0.10	0.57	0.32
Lysine biosynthesis	0.79 ± 0.04^a^	0.75 ± 0.03^b^	0.75 ± 0.018^b^	0.77 ± 0.02^ab^	0.32	0.20	0.06
Energy metabolism	0.79 ± 0.04	0.77 ± 0.06	0.78 ± 0.01	0.78 ± 0.03	0.10	0.51	0.85
Fructose and mannose metabolism	0.73 ± 0.04	0.74 ± 0.02	0.74 ± 0.03	0.75 ± 0.05	0.01	1.00	0.61
Nitrogen metabolism	0.67 ± 0.04	0.68 ± 0.02	0.68 ± 0.018	0.68 ± 0.01	0.10	0.04	0.32
Translation factors	0.62 ± 0.02^a^	0.61 ± 0.02^ab^	0.60 ± 0.015^b^	0.61 ± 0.02^ab^	0.31	0.40	0.10
Bacterial secretion system	0.58 ± 0.01	0.59 ± 0.04	0.59 ± 0.033	0.59 ± 0.04	0.09	0.77	0.14
Nucleotide excision repair	0.48 ± 0.01^a^	0.47 ± 0.01^ab^	0.46 ± 0.015^b^	0.47 ± 0.01^b^	0.65	0.52	0.03
Pentose and glucuronate interconversions	0.42 ± 0.01^a^	0.41 ± 0.01^b^	0.41 ± 0.02^b^	0.41 ± 0.02^b^	0.68	0.30	0.10
Selenocompound metabolism	0.36 ± 0.02	0.35 ± 0.01	0.36 ± 0.013	0.35 ± 0.01	0.10	0.08	0.18
Folate biosynthesis	0.33 ± 0.02	0.35 ± 0.03	0.35 ± 0.033	0.35 ± 0.02	0.02	0.17	0.26
RNA polymerase	0.24 ± 0.03	0.22 ± 0.03	0.21 ± 0.01	0.21 ± 0.01	0.07	0.21	0.11
C5-Branched dibasic acid metabolism	0.21 ± 0.05	0.21 ± 0.02	0.22 ± 0.015	0.22 ± 0.03	0.06	0.42	0.45
D-Glutamine and D-glutamate metabolism	0.17 ± 0.00^a^	0.17 ± 0.00^a^	0.16 ± 0.00^b^	0.17 ± 0.01^a^	0.46	0.88	0.10
Feces							
Carbohydrate digestion and absorption	0.031 ± 0.01	0.026 ± 0.01	0.027 ± 0.01	0.027 ± 0.01	0.60	0.07	0.49
Nucleotide metabolism	0.070 ± 0.01	0.069 ± 0.01	0.072 ± 0.01	0.071 ± 0.01	0.03	0.60	0.27
Bacterial chemotaxis	0.398 ± 0.02	0.431 ± 0.03	0.404 ± 0.04	0.389 ± 0.02	0.17	0.10	0.31

MYCOT = effect of mycotoxin contamination diets as control or Mycotoxins evaluated by a Friedman test. YCWE = effect of the addition or not of an adsorbent in the diet evaluated by a Friedman test. M × Y = Effect of mycotoxin contamination and addition of an adsorbent interaction evaluated by a Kruskal-Wallis test. ^a-c^ Means 62 followed by different letters differ at *p* ≤ 0.10.

Relative abundance of Lysine biosynthesis tended to be higher (*p* = 0.06) in the CTRL YCWE− treatment and lower in the CTRL YCWE+ and MYCOT YCWE− treatments. In this sense, the MYCOT YCWE+ treatment was similar to the CTRL YCWE− treatment. Nucleotide excision repair was higher in CTRL YCWE− and lower in treatments with addition of MYCOT (*p* = 0.03). D-Glutamine and D-glutamate metabolism tended to be lower (*p* = 0.10) in the MYCOT YCWE− treatment than in the other treatments. The abundance of the signaling mechanism Two-component system metabolic pathway was higher (*p* = 0.04) in the MYCOT YCWE− treatment and lower in the CTRL YCWE− treatment. Secretion system was more abundant (*p* = 0.02) in treatments with addition of MYCOT and lower in CTRL YCWE− treatment.

Mycotoxin contamination tended to reduce the predicted abundance of metabolic pathways related to Transporters (*p* = 0.10), DNA repair and recombination proteins (*p* = 0.08), and RNA polymerase (*p* = 0.07), while showing a tendency to increase predictions for Bacterial motility proteins (*p* = 0.10), the Bacterial secretion system (*p* = 0.09), and C5-branched dibasic acid metabolism (*p* = 0.06). In addition, MYCOT increased the abundance of pathways involved in Fructose and Mannose metabolism (*p* = 0.01) and Folate biosynthesis (*p* = 0.02). In the rumen, YCWE supplementation was associated with greater nitrogen metabolism (*p* = 0.04) and a tendency toward reduced Selenocompound metabolism (*p* = 0.08).

In feces (Table 6), contamination with MYCOT resulted in greater Nucleotide metabolism (*p* = 0.03) and there was a trend in YCWE towards lower Carbohydrate digestion and absorption (*p* = 0.07) and greater Bacterial chemotaxis (*p* = 0.10).

## 4 Discussion

Hypotheses about the effects of several mycotoxins in modifying the ruminal microbiota due to their antimicrobial activity have been postulated to explain effects observed on rumen functions, such as decreased fermentative capacity, altered fermentation product profile and lower digestibility (Gallo et al., 2015, 2020; Jiang et al., 2019; Batista et al., 2024). According to our knowledge, this is the first study to demonstrate how the contamination of cattle diet with multiple mycotoxins at relevant concentrations under practical conditions, and the use of an organic adsorbent based on yeast cell walls and algae polysaccharides could alter the ruminal and fecal microbial community, and its predicted metabolic functionality.

The greater richness indices of the ruminal microbial population with mycotoxins demonstrates that contamination can affect the naturally predominant microbial taxa for the type of diet used, allowing the growth of other taxa in the ruminal environment, thus increasing richness indices. Lower richness of microbiome gene content and taxa was tightly linked to higher feed efficiency in dairy cows (Shabat et al., 2016). Also, the higher diversity indices observed in this study with MYCOT contamination may be associated with lower efficiency in the use of energy from the diet by microorganisms and lower feed efficiency, as observed by Shabat et al. (2016), where higher diversity indices resulted in greater energy loss associated with null or inefficient routes (such as methane production) and consequently lower feed efficiency. These findings may help explain the lower feed efficiency observed in finishing Nellore bulls fed a diet contaminated with mycotoxins, without the use of an adsorbent based on yeast cell walls (Custodio et al., 2020).

Moreover, the increased microbial richness associated with mycotoxin contamination likely contributes to higher nitrogen excretion via urine and reduced nitrogen retention in the animal’s body, as reported by Batista et al. (2024). In Nellore cattle, lower nitrogen retention was directly associated with greater ruminal microbial richness (Alves et al., 2021). In contrast to the rumen environment, contamination with deoxynivalenol (DON; 0, 2.5, 5, and 10 mg/kg diet) in the cecal compartment of broiler chickens reduced both richness and diversity indices (Lucke et al., 2018). Traditionally, high microbial diversity has been considered indicative of a more stable microbiota, helping to prevent pathogen colonization (Han et al., 2017). However, alternative perspectives suggest that a less diverse but more specialized bacterial community may exploit limiting resources more efficiently, thereby enhancing host energy acquisition (Lozupone et al., 2012; Siegerstetter et al., 2017).

The effects of YCWE supplementation on richness and diversity indices were independent of MYCOT contamination, as no MYCOT × YCWE interaction was detected. In this sense, although the use of YCWE reduced richness (ACE and Chao 1 index), it acted in a similar way to MYCOT by increasing microbial diversity indices. In contrast, the Shannon index remained unchanged, indicating that YCWE supplementation did not affect the overall richness–evenness structure (entropy) of the ruminal microbiota, even though shifts in specific taxa were observed.

Overall, even modest shifts at several taxonomic levels could affect the ruminal fermentation process. For example, the trend toward greater relative abundance of the Clostridia class in animals fed MYCOT suggests that contamination attenuated other microbial groups, such as *Christensenellaceae* family, and improved the conditions for the growth of microorganisms of Clostridia class. Clostridia have several groups of human and animal pathogens (Cruz-Morales et al., 2019). Additionally, within this group there are several ammonia hyperproducing bacteria, which could be associated with greater N excretion in urine (Batista et al., 2024). In this sense, the Lachnospiraceae family, more abundant in the MYCOT diet, was previously directly related to Nellore steers with lower N retention (Alves et al., 2021). The mycotoxin contamination also promoted the enrichment of *Dialister* in the rumen. This genera has been associated with lower feed efficiency in beef cattle (Li and Guan, 2017) and may be correlated to the lower feed efficiency observed in beef cattle fed a diet contaminated with mycotoxins by Custodio et al. (2020).

Contamination with MYCOT similarly reduced the relative abundance of important cellulolytic bacterial groups. This was evidenced by the decrease observed in the rumen for the *Christensenellaceae* family and the lower abundance found in feces for the Succinivibrionaceae family, as well as the genus *Pseudobutyrivibrio, Streptococcus, Prevotellaceae_Ga6A1* and Bacteroidales_RF16_group. These changes may also contribute to the understanding of the lower digestibility of NDF reported by Batista et al. (2024). In this sense, in culture medium, May et al. (2000) demonstrated antimicrobial activity of fumonisins against *Ruminococcus albus* and *Methanobrevibacter ruminantium*. Contamination of chicken diets with DON also reduced the relative abundance of a *Ruminococcus* taxon in the chicken cecum (Metzler-Zebeli et al., 2020). Therefore, it indicates that MYCOT likely had an antimicrobial effect in the rumen on fiber-degrading bacteria.

Although this study highlights changes in the structure of the ruminal microbial community with MYCOT contamination, the total production of volatile fatty acids (VFA) in the rumen and the proportion of predominant VFAs (acetate, propionate, butyrate) were not affected by the contamination (Batista et al., 2024). This highlights in some ways the redundancy during key steps of anaerobic feed degradation within the rumen (Söllinger et al., 2018). In other words, even with changes in the microbiota profile, the main fermentation products are maintained. The MYCOT × YCWE interaction effects observed by Batista et al. (2024) on the proportion of isovalerate and valerate and the effect of MYCOT and YCWE on the proportion of isobutyrate, may be related to the taxa abundance differences associated in the present study with MYCOT × YCWE interaction. Suggesting that these changes in the rumen microbiota alter the profile of degraded and fermented amino acids in the rumen. However, contrary to our hypothesis, in many cases the use of YCWE in the MYCOT diet did not result in relative abundances similar to the CTRL YCWE− treatment.

The MYCOT × YCWE interaction affected the relative abundance of several families and genera in the rumen. Overall, these patterns suggest that mycotoxin contamination combined with YCWE supplementation reshaped specific microbial niches instead of inducing uniform shifts across the rumen microbiota. For example, the *Subdoligranulum* ruminal abundance was higher in the rumen of animals fed MYCOT YCWE+ compared to animals fed diets without MYCOT. This genus is a strictly anaerobic, butyrate-producing, gram-negative bacterium (Holmstrøm et al., 2004) and its role in the rumen has not been well studied. Conversely, the enrichment of well-known bacterial plant pathogens, such as the Pectobacteriaceae family, and its genus *Dickeya* (Mansfield et al., 2012) in the rumen of animals that fed MYCOT YCWE− may be associated with the growth of opportunistic plant-associated bacteria, possibly due to reduced competitive pressure from fibrolytic bacteria previously discussed, or altered immune parameters, as reported by Batista et al. (2024). Additionally, *Enterococcus* were enriched in the rumen of animals fed MYCOT YCWE−. This suggests a shift towards bacterial groups that are commonly found in the gastrointestinal systems of cattle. However, these bacteria can act as opportunistic pathogens in conditions of stress or when the immune system is compromised (Cebeci, 2024).

In the rumen, with the use of YCWE adsorbent, a greater number of genera had their relative abundance differentially affected in relation to MYCOT contamination. The lower relative abundance observed with the use of YCWE may be associated with the trend towards a lower proportion of acetate with the use of YCWE observed in the animals in this study (Batista et al., 2024). The decrease in the proportion of acetate with the use of YCWE probably resulted in an evident numerical decrease in the relative abundance of total Archaea in the rumen and a significant reduction in the genus *Methanomicrobiaceae*. This also suggests that lower methanogenesis in the ruminal environment may occur with YCWE. However, studies measuring methane production are necessary to analyze this hypothesis. Indeed, the lower abundance of *Succinivibrio* observed with YCWE also reinforces this hypothesis, since the production of formate by Succinivibrio sp. may be a factor in the rate of methanogenesis in the rumen (O’Herrin and Kenealy, 1993; Wallace et al., 2015) depending on the availability of CO_2_. Zhou et al. (2023) demonstrated the ability of an additive based on yeast cell-wall polysaccharides to modulate gut microbial composition, by enhancing the proliferation of beneficial microbes and suppression of pathogens colonization in the gut in laying hens. According to Liu et al. (2021), the main components of yeast cell-wall polysaccharides can adhere to pathogenic bacteria in the gut and modulate gut microbiota and intestinal integrity.

In evaluating the metabolic pathways of the rumen microbiota, we observed that MYCOT contamination and the use of YCWE affected metabolic pathways directly linked to protein synthesis. However, the use of YCWE in the contaminated diet partially recovered some responses, such as purine and pyrimidine metabolism, as well as the lysine biosynthesis and translation factors. Purines and pyrimidines are indispensable components for the synthesis of DNA and RNA, therefore, necessary for cell division and consequent microbial growth, as they provide the essential nucleotides required for genome replication and protein synthesis (Watson and Crick, 1953). Lysine biosynthesis, an essential amino acid necessarily present in microbial protein (Sok et al., 2017), and protein translation factors. The tendency for lower abundance of the RNA polymerase metabolic pathway with MYCOT suggests that contamination potentially reduced the ability of microbial cells to multiply, by reducing the ability to duplicate genetic material. However, we can observe that the use of adsorbent in the diet without the addition of mycotoxins, in the same way as MYCOT contamination, impaired metabolic pathways related to microbial protein synthesis, such as the ribosome metabolic pathways, aminoacyl-tRNA biosynthesis, translation proteins. It is known that aminoacyl-tRNA biosynthesis is a crucial process in the translation of genetic information for protein synthesis, which involves the attachment of amino acids to their corresponding tRNA molecules, forming aminoacyl-tRNAs (King, 2007). And this protein translation process takes place in ribosomes. It highlights the necessity of studies directly evaluating the flow of microbial protein through the bovine duodenum and the amino acid composition of the microbial mass to understand the effects of MYCOT on microbial protein synthesis in the rumen.

While lower abundance of metabolic pathways linked to protein synthesis were found with MYCOT, contamination resulted in greater abundance of metabolic pathways linked to the defense activities of microorganisms. For example, two-component system and secretion system were higher in MYCOT YCWE– and lower in CTRL YCWE–. The two-component system is a fundamental signaling mechanism that allows bacteria to sense and respond to changes in their external environment (Hirakawa et al., 2020). These systems have been proposed as potential targets for the development of antibacterial agents (Hirakawa et al., 2020). This pattern indicates that the microbiota would be acting with defenses against mycotoxins. Mycotoxins are fungal secondary metabolites whose toxicity can trigger rumen microbial adaptation and detoxification processes (Fink-Gremmels, 2008), but activation of stress responses such as two-component signaling and secretion systems may divert energy from bacterial growth (Zhu and Dai, 2024), reducing fibrolytic activity and protein synthesis efficiency in the rumen.

Minor effects of treatments were observed on the fecal microbiome, mainly reflected in the limited number of metabolic pathways affected. Unlike in the rumen, fecal microbial richness indices were not influenced by contamination or adsorbent use; however, contamination with MYCOT reduced ruminal bacterial diversity, as indicated by lower Shannon entropy and Simpson indices, leading to a community dominated by fewer groups (Lozupone et al., 2012). These differences in diversity indices are possibly associated with shifts in the relative abundance of specific groups, such as reduction in the abundance of Gammaproteobacteria class, as well as the families p-251-o5, Streptococcaceae and Succinivibrionaceae. Additionally, the abundance of the genus *Megasphaera*, *Bacteroidales_RF16_group*, *Pseudobutyrivibrio, Streptococcus, Subdoligranulum, Roseburia,* and *Prevotellaceae_Ga6A1* has also decreased. The modest impact of MYCOT on the fecal microbiome is likely related to the rumen microbiota’s capacity to biotransform mycotoxins (Loh et al., 2020), or to the absorption of mycotoxins through the gastrointestinal tract before they reach the large intestine (Grenier and Applegate, 2013). Thus, at the fecal level, the ability of the rumen microbiota to convert certain mycotoxins into less or non-toxic derivatives (Fink-Gremmels, 2008; Gallo et al., 2015; Loh et al., 2020), may help mitigate their effects on the fecal microbiome.

Conversely, supplementation with YCWE increased fecal bacterial diversity by the Shannon index, indicating a partial restoration of richness in the gut microbiome. The Shannon index is more sensitive to the number of OTUs, whereas Simpson is more influenced by evenness, such as dominance patterns (Hopton et al., 2017). This suggests that YCWE may have promoted the abundance of additional bacterial groups rather than simply redistributing abundance evenly. This effect is probably due to the dual role of YCWE, the ability to bind mycotoxins, thereby reducing selective pressure on sensitive taxa, and their provision of fermentable substrates that support microbial proliferation (Yiannikouris et al., 2006; Jouany, 2007; Zhou et al., 2023). In this context, YCWE supplementation increased the abundance in feces of Morganellaceae family and the genus *Christensenellaceae_R-7*, *Clostridia_UCG-014,* and *F082*.

## 5 Conclusion

This study demonstrated that contamination of beef cattle diets with multiple mycotoxins induced shifts in the ruminal microbial community, reducing the relative abundance of key cellulolytic bacteria within the Christensenellaceae family, while simultaneously altering the abundance of microorganisms from the Clostridia class and the Lachnospiraceae family. Mycotoxin contamination also altered microbial richness and diversity indices, which have been linked to reduced efficiency of dietary energy utilization, while simultaneously decreasing metabolic pathways potentially associated with impaired microbial growth and protein synthesis. In contrast, supplementation with yeast cell wall extract (YCWE) effectively mitigated several of these adverse effects and partial recovery of disrupted metabolic pathways. In addition, YCWE supplementation reduced the abundance of several genera, particularly *Succinivibrio* and *Methanomicrobium*, which may be associated with a reduction in methanogenesis activity in the rumen. The novelty of this work lies in providing direct evidence that YCWE supplementation can counteract the microbiome disruptions caused by dietary mycotoxins in cattle, highlighting its role as a preventive nutritional strategy. While previous studies have reported performance and health benefits of YCWE, our findings identify the microbial groups most responsive to YCWE and quantify its contribution to preserving rumen functionality. From a practical perspective, incorporating YCWE into ruminant diets contaminated with mycotoxins offers a feasible approach to reduce the risk of fermentation inefficiencies associated with microbial dysbiosis due to mycotoxin contamination.

## Data Availability

The 16S rRNA gene amplicon sequencing data (Accession Nº PRJNA1334244) used in this study have been stored in the Sequence Read Archive (SRA) database of the NCBI. The records can be accessed through the following link: https://www.ncbi.nlm.nih.gov/sra/PRJNA1334244.

## Footnotes

^1^ https://github.com/vsbuffalo/scythe
